# ALK‐positive lung cancer diagnosed with abdominal lymph nodes in a patient receiving hemodialysis

**DOI:** 10.1111/1759-7714.13205

**Published:** 2019-10-07

**Authors:** Sung H. Song, Ji W. Ryu, Hye Y. Jwa, Chang W. Ha, Hyun Kim, Jae M. Jo, Sang H. Han

**Affiliations:** ^1^ Department of Internal Medicine Cheju Halla General Hospital Jeju Republic of Korea; ^2^ Department of Pathology Cheju Halla General Hospital Jeju Republic of Korea; ^3^ Department of Radiology Cheju Halla General Hospital Jeju Republic of Korea; ^4^ Department of Internal Medicine Jeju National University School of Medicine Jeju Republic of Korea

**Keywords:** ALK, cholestasis, hemodialysis, lung, lymph node

## Abstract

There is little consensus in the literature about the administration of crizotinib in patients with end‐stage renal disease undergoing hemodialysis. A 69‐year‐old male patient, who was receiving regular hemodialysis due to end‐stage renal disease, visited the hospital with symptoms of repeated abdominal pain. There was no suspicious finding of cancer within the thorax. After biopsy, the abdominal lymph nodes were identified as adenocarcinoma originating from the lung following computed tomography (CT) scan, and ALK rearrangement was confirmed. The patient achieved a partial response following the administration of crizotinib, although treatment was discontinued because of unknown cholestasis. Overall survival was eight months. Although crizotinib has clear efficacy in patients with ALK‐positive lung cancer with end‐stage renal disease, the optimal dose of crizotinib should be identified in patients receiving regular hemodialysis.

## Introduction

Cancer metastasis in the surrounding organs is often accompanied by lymph node (LN) enlargement.^1^, ^2^ In this case, the patient visited the hospital with repeated abdominal pain and cancer was not diagnosed within the thoracic cavity. Abdominal LN enlargement was revealed by computed tomography (CT) scan. After biopsy, the abdominal LNs were identified as adenocarcinoma originating from the lung, and ALK rearrangements were also found. In addition, the patient was undergoing hemodialysis due to end‐stage renal failure. Until now, there is little consensus in the literature on the administration of crizotinib in patients with end‐stage renal disease undergoing hemodialysis. Here, we report our experience and findings.

## Case report

**Figure 1:**
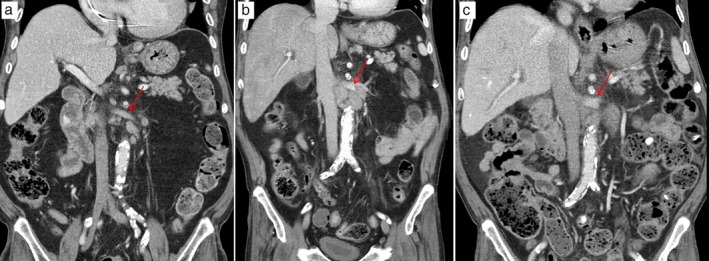
Abdominal computed tomographic (CT) scans. (**a**) The abdominal CT scan showed multiple small lymph nodes at the gastric cardia, on the left gastric artery, and along the paraaortic lymphatic chains. (**b**) After three months, enlargement of lymph nodes could be seen, especially para‐aortic lymph nodes at the level of the left renal vein. (**c**). After one month of crizotinib treatment, para‐aortic lymph nodes were markedly regressed. *arrows, left renal vein.

A 69‐year‐old man presented to our hospital with epigastric pain. He had been diagnosed with end‐stage renal disease (ESRD) from diabetic nephropathy eleven years previously and was on hemodialysis. Abdominal computed tomography (CT) was performed and revealed multiple small lymph nodes at the gastric cardia, on the left gastric artery, and along the para‐aortic lymphatic chains (Fig 1a). Gastric endoscopy was performed to consider the pathologies in the surrounding organs and a peptic ulcer with hemorrhage was detected. The patient was discharged following treatment.

**Figure 2:**
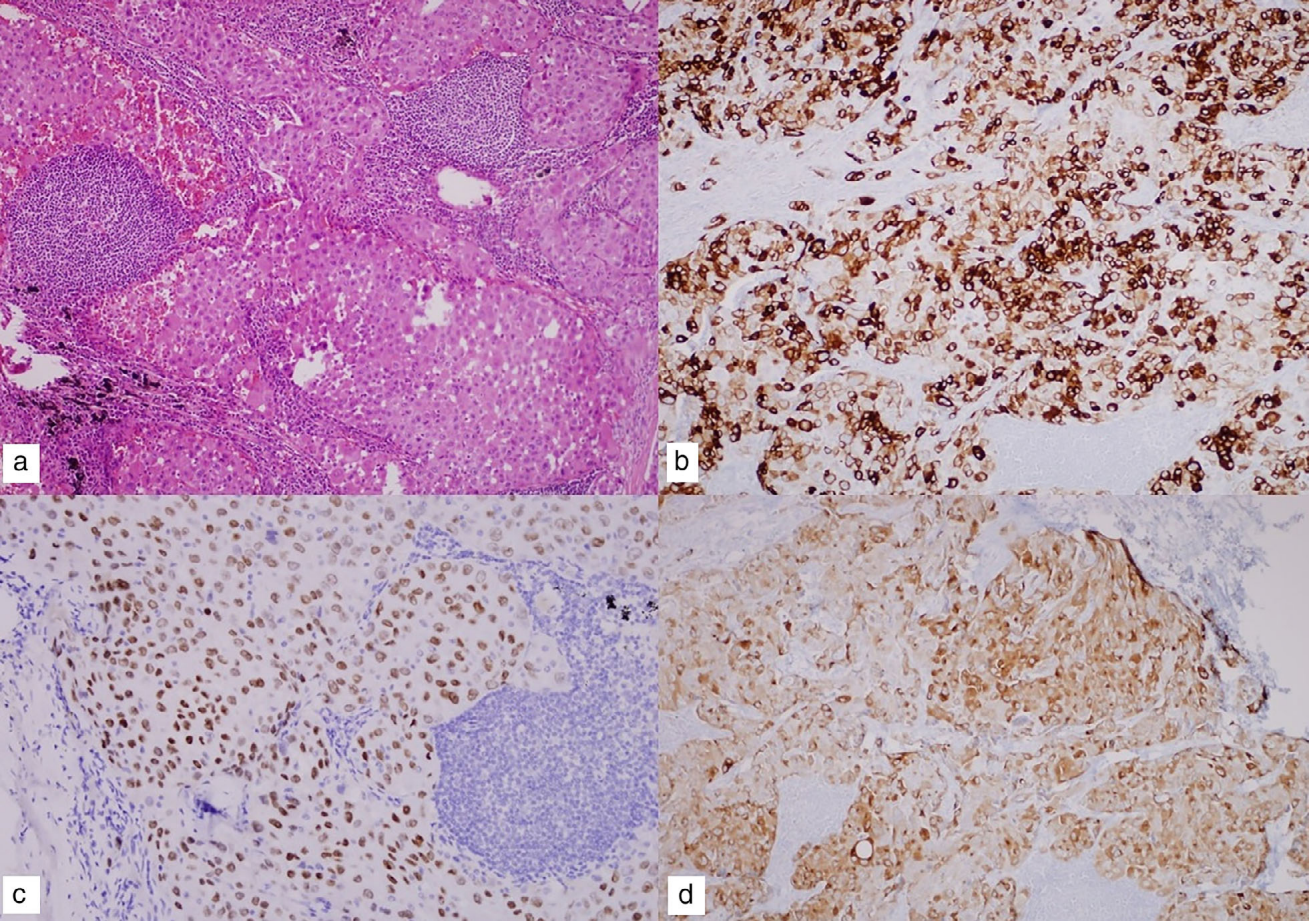
Pathological findings. The tumor showed undifferentiated malignant neoplasm of uncertain origin ([**a**] hematoxylin and eosin, 100x). The tumor cells were positive for CK‐7 ([**b**] immunohistochemistry, 100x), TTF‐1 ([**c**] immunohistochemistry, 200x), and ALK ([**d**] immunohistochemistry, 200x).

**Figure 3:**
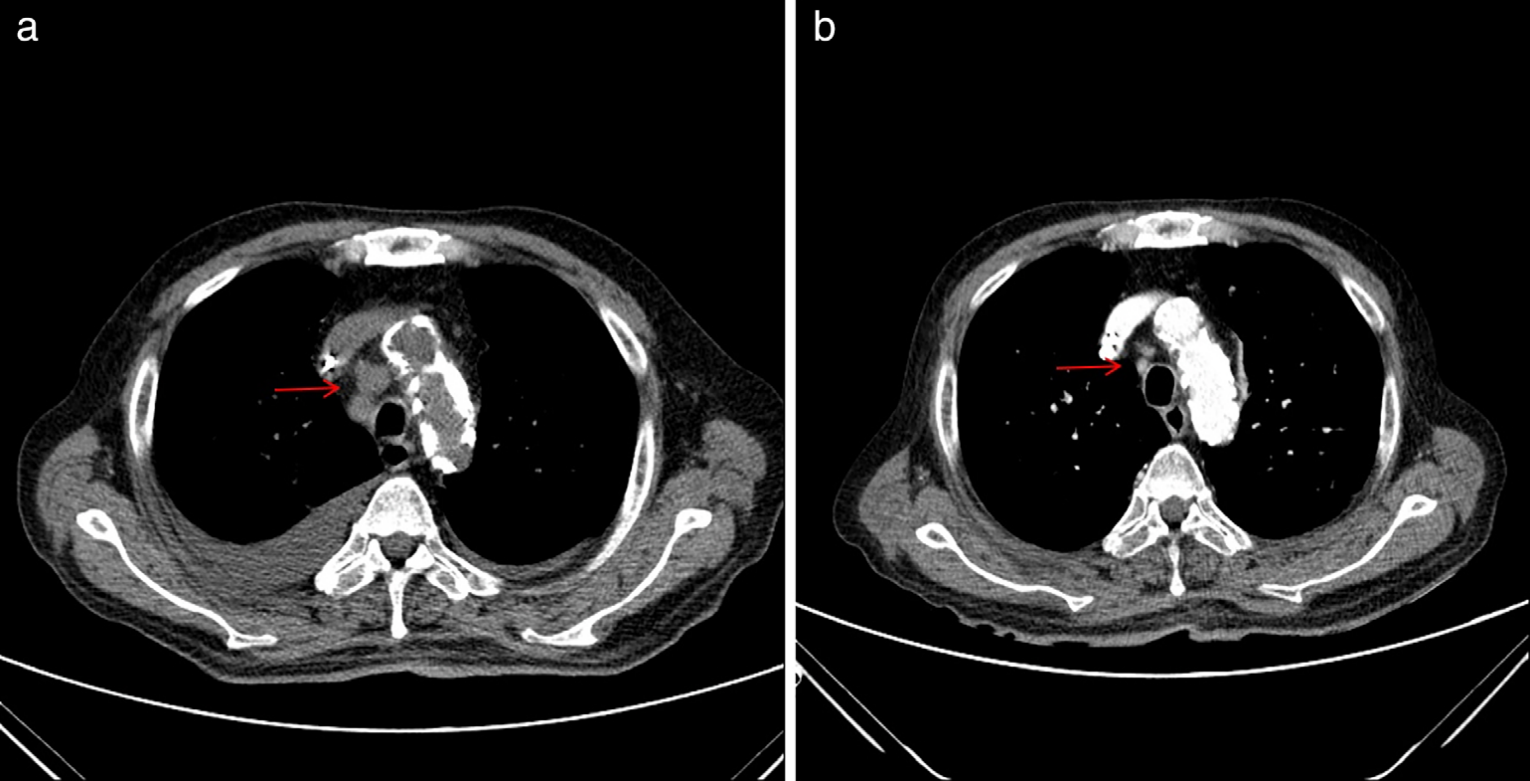
Chest computed tomographic (CT) scans. (**a**) The chest CT scan showed enlargement of multiple mediastinal lymph nodes. (**b**) After one month of crizotinib treatment, mediastinal lymph nodes were found to be markedly regressed.

**Figure 4:**
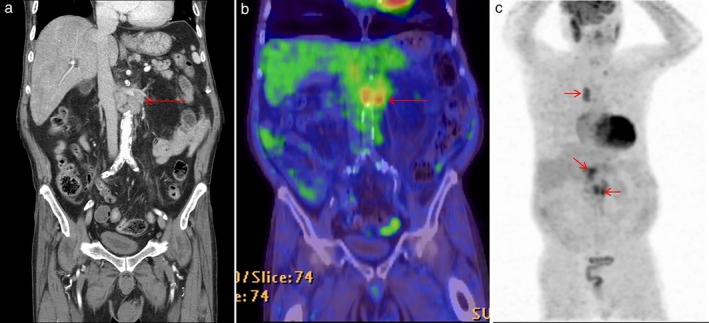
Positron emission tomography‐computed tomography (PET‐CT) scans. (**a**, **b**) Enlarged para‐aortic lymph nodes revealed hypermetabolic activity on PET‐CT. (**c**) Maximum intensity projection (MIP) image in PET‐CT showed no definite hypermetabolic mass in the chest and abdomen, except mediastinal and abdominal lymph nodes (arrows).

However, four months later, the patient was readmitted to our hospital suffering with abdominal pain. Abdominal CT detected an increase in size of the previously visible LNs (Fig 1b). We performed a laparoscopic surgical biopsy to confirm the exact histopathological findings which revealed an undifferentiated malignant neoplasm of uncertain origin (Fig 2a). CK7, TTF‐1, and napsin A were positive in the immunochemical test; LCA, PSA, CDX2, CK20, S‐100, and HMB45 showed negative results (Table 1). The lesion was subsequently diagnosed as poorly‐differentiated adenocarcinoma originating from the lung (Fig 2b,c). In addition, ALK IHC was positive (Fig 2d) and ALK translocation t(2:5), detected in 45% of ALK by FISH, was also found to be positive. On the chest CT, mediastinal LN enlargement was observed (Fig 3a). However, there was no evidence of lung cancer, such as mass or nodule in the lung parenchyma. PET‐CT showed FDG high uptake in the abdominal, mediastinal, and left supraclavicular LNs (Fig 4). Treatment with Crizotinib, an ALK inhibitor, was initiated.

**Table 1:** Immunohistochemistry results

Immunohistochemistry	Finding
Pan‐CK	+
CK 7	+
TTF‐1	+
Napsin A	+
LCA, CD30, CD3, CD5	−
PSA	−
CDX2, CK20	−
CD31, factor VIII	−
S‐100, HMB45	−
Calretinin, CK5/6, WT1	−
HAS	−

+, stained; −, unstained.

Without any guideline for the administration of crizotinib in ESRD patients undergoing hemodialysis, 250 mg once daily was administered as a dose corresponding to that given in severe renal disease patients.^3^, ^4^ No significant adverse events occurred during the crizotinib treatment period, except for mild generalized edema. On the chest and abdominal CT performed one month after the initiation of treatment, the size of the enlarged LNs showed a partial response (PR) (Fig 1c, 3b).

After seven months of treatment with crizotinib, the patient visited the emergency room again with right upper abdominal pain. Laboratory investigations revealed elevated total bilirubin level of 10.75 mg/dL, direct bilirubin level of 10.14 mg/dL, CRP level of 6.44 mg/dL, and procalcitonin level of 1.89 ng/mL. Abdominal CT showed intrahepatic bile duct dilatation without any definite obstructive lesion. Initially, treatment was started following the diagnosis of cholangitis. Crizotinib was continued, and the patient was discharged following pain relief. About one month later, he suddenly developed jaundice and was hospitalized again. His total bilirubin level had risen to 17.89 mg/dL and crizotinib was subsequently discontinued. However, as his heart failure worsened after hospitalization, systemic and pulmonary edema were aggravated, and the patient expired following a sudden cardiac arrest.

## Discussion

In the case described here, following immunohistochemical (IHC) staining, the patient was finally diagnosed with adenocarcinoma of the lung with positive ALK and FISH.^5^ There were no severe adverse effects observed, except edema which is a relatively common finding in a phase 3 study of crizotinib.^6^ Meanwhile, he had been hospitalized for cholangitis at seven months after crizotinib treatment. The total bilirubin and direct bilirubin levels were elevated without any fever or severe leukocytosis. Abdominal CT showed intrahepatic bile duct dilatation without any definite obstructive lesion. Although cholangitis was not clear, other etiologies were difficult to consider, and therefore, treatment was commenced with empirical antibiotics. Although acute hepatitis has been reported to be associated with crizotinib therapy,^7^ cholangitis or cholestasis have not previously been described. The other type of ALK inhibitor, ceritinib, has been reported to cause cholestasis with intrahepatic bile duct dilatation similar to our case.^8^ The patient reported here died suddenly from cardiac arrest. Therefore, it was not possible to determine exactly whether the cause of death was due to crizotinib‐associated cholestasis along with intrahepatic bile duct dilatation. However, considering the fact that this disorder occurred during the use of a similar class of drug, ceritinib, it is not possible to exclude the possibility that crizotinib caused cholestasis along with intrahepatic bile duct dilatation.

It has been reported that patients with stage 4 NSCLC with ALK rearrangement have an average survival time of 6.8 years when treated with an ALK inhibitor.^9^ In this case, the patient had underlying cardiac failure and ESRD and because he was on hemodialysis, the prognosis was expected to be somewhat worse than that in patients in general. Molecular genetic testing of lung cancer tissues alone led us to conclude that the prognosis was good. Nevertheless, our study concludes that patients with severe underlying disease, such as heart failure or ESRD, may not have a good prognosis.

## Disclosure

The authors report no conflict of interest.
